# Susceptibility of Domestic Swine to Experimental Infection with Severe Acute Respiratory Syndrome Coronavirus 2

**DOI:** 10.3201/eid2701.203399

**Published:** 2021-01

**Authors:** Brad S. Pickering, Greg Smith, Mathieu M. Pinette, Carissa Embury-Hyatt, Estella Moffat, Peter Marszal, Charles E. Lewis

**Affiliations:** University of Manitoba, Winnipeg, Manitoba, Canada (B.S. Pickering);; Canadian Food Inspection Agency, Winnipeg (B.S. Pickering, G. Smith, M.M. Pinette, C. Embury-Hyatt, E. Moffat, P. Marszal);; Iowa State University, Ames, Iowa, USA (B.S. Pickering, C.E. Lewis)

**Keywords:** respiratory infections, severe acute respiratory syndrome coronavirus 2, SARS-CoV-2, SARS, COVID-19, coronavirus disease, zoonoses, viruses, coronavirus, pandemics, One Health, public health, antibodies, livestock, swine

## Abstract

Severe acute respiratory syndrome coronavirus 2 (SARS-CoV-2), the agent that causes coronavirus disease, has been shown to infect several species. The role of domestic livestock and associated risks for humans in close contact with food production animals remains unknown for many species. Determining the susceptibility of pigs to SARS-CoV-2 is critical to a One Health approach to manage potential risk for zoonotic transmission. We found that pigs are susceptible to SARS-CoV-2 after oronasal inoculation. Among 16 animals, we detected viral RNA in group oral fluids and in nasal wash from 2 pigs, but live virus was isolated from only 1 pig. Antibodies also were detected in only 2 animals at 11 and 13 days postinoculation but were detected in oral fluid samples at 6 days postinoculation, indicating antibody secretion. These data highlight the need for additional livestock assessment to determine the potential role of domestic animals in the SARS-CoV-2 pandemic.

Severe acute respiratory syndrome coronavirus 2 (SARS-CoV-2) causes coronavirus disease (COVID-19) in humans; symptoms can range from asymptomatic to mild or severe, including severe respiratory distress and sometimes death (*1*). Rapidly spreading, the novel SARS-CoV-2 virus emerged in China in late 2019, and on March 11, 2020, the World Health Organization declared a pandemic (*2*). SARS-CoV-2 is believed to have originated in bats, but its origins are still under intense investigation, and reports continue to identify the ability of the virus to infect additional animal species (*3*–*8*).

Detection of natural infections sheds light on knowledge gaps in SARS-CoV-2 transmission and raised concerns of amplifying or reservoir hosts. In turn, clarification of wild and domestic animal susceptibility can help us assess their potential roles in and risks for transmission to prevent future disease spread. Domestic swine, one of the most highly produced agricultural species, previously have impacted public health (*9*–*12*). Backyard, small stakeholder animal production has increased in both rural and urban environments and provides a source of high-quality protein and income in these areas, but the practice also can serve as a source for zoonotic disease; therefore, the potential role of pigs in the spread of SARS-CoV-2 should be investigated (*13*). Recent evidence for involvement of production animals in SARS-CoV-2 transmission was highlighted in the Netherlands, where anthroponotic transmission from humans to farmed mink was proposed with subsequent zoonotic transmission to >2 humans from mink (*14*). That case further exemplifies the need to identify the potential role of production animals in disease transmission.

Angiotensin-converting enzyme 2 (ACE2) has been identified as the receptor for SARS-CoV-2 in human cells (*15*). A BLAST (https://blast.ncbi.nlm.nih.gov) query of the protein database by using translated nucleotide (BLASTx) from the human ACE2 coding sequence predicts 98% coverage and 81% identity for the homologous receptor in swine. Of note, using the same search, mink (*Mustela lutreola*) show 82% similar identity and domestic felines (*Felis catus*) show 85% similar identity to the human ACE2 for their cognate receptors. Moreover, mink and cats both have been reported to be susceptible to SARS-CoV-2 and have shown transmission to other animals (*5*,*16*). Zhou et al. (*17*) used in vitro infectivity studies testing ACE2 receptors from laboratory mice, horseshoe bats (*Rhinolophus sinicus*), civets, and the domestic pig, and found all receptors except those from mice entered HeLa cells, indicating a functional target for SARS-CoV-2. Moreover, the authors used additional known coronavirus receptors, including both aminopeptidase N and dipeptidyl peptidase 4, and found neither are used for cell entry (*17*), underlining the specificity for the ACE2 receptor.

We aimed to determine whether domestic swine are susceptible to SARS-CoV-2 infection by testing for live SARS-CoV-2 virus after experimental inoculation. After oronasal inoculation, we assessed swine for clinical signs and pathology, evidence of virus shedding, viral dissemination within tissues, and seroconversion.

## Methods

Experimental design, including housing conditions, sampling regimens, and humane endpoints, were approved by the Animal Care Committee of the Canadian Science Centre for Human and Animal Health (no. AUD #C-20-005). All procedures and housing conditions were in strict accordance with the Canadian Council on Animal Care guidelines. Group housing was in Biosecurity Level 3 (BSL-3) zoonotic large animal cubicles. Animals were provided commercial toys for enrichment and access to food and water ad libitum. All invasive procedures, including experimental inoculation and sample collection (nasal washes, rectal swabs, and blood collection), were performed under isoflurane gas anesthesia, and animals were euthanized by intravenous administration of a commercial sodium pentobarbital solution.

### Study Design

We obtained 19 domestic, 8-week-old American Yorkshire crossbred pigs (*Sus scrufa domesticus*), 6 castrated males and 13 females, locally sourced from a high health status farm in Manitoba, Canada. We obtained animals locally, rather than from a specific pathogen–free colony, to determine the risk to farmed pigs in Canada. We oronasally challenged 16 pigs with 1 × 10^6^ PFU/animal in a total of 3 mL Dulbecco’s Modified Eagle’s Medium (DMEM; Wisent, http://www.wisentbioproducts.com) under sedation with isoflurane. We distributed 1 mL per nostril and placed 1 mL in the distal pharynx by using a sterile, tomcat-style catheter. We confirmed the challenge dose by back-titration of the inoculum on Vero E6 cells.

We divided the 16 inoculated pigs into 2 groups of 8, and each group was housed in a separate BSL-3 cubicle. At day 10, we introduced 2 naive pigs, 1 into each cubicle with the inoculated pigs, to serve as in-room transmission controls. Animal numbers were not based on power analysis but on limitations of the containment animal room size and requirements of Canadian Council on Animal Care guidelines. Group assignments for day of euthanasia and necropsy were based on randomization at the time of permanent animal identification via ear tags.

At the time of inoculation (day 0) and every other day beginning at 3 days postinoculation (dpi) until day 15, we performed a physical examination, including collection of blood; rectal, oral, and nasal swabs; and nasal wash with sterile Delbecco’s phosphate-buffered saline (D-PBS). We began performing necropsies and post-mortem sampling starting at 3 dpi (Table 1). We sampled and necropsied 1 additional uninoculated pig to serve as a farm control providing negative control tissues. We sampled the remaining pigs at 22 dpi and 29 dpi. We collected group oral fluids from rope chews daily.

**Table 1:** Sampling and necropsy schedule for pigs experimentally inoculated with SARS-CoV-2*

Pig ID	Days post inoculation									
	0	3	5	7	9	11	13	15	22	29
Cubicle 1										
20–01	S	**S**	–	–	–	–	–	–	–	–
20–02	S	S	**S**	–	–	–	–	–	–	–
20–03	S	S	S	**S**	–	–	–	–	–	–
20–04	S	S	S	S	**S**	–	–	–	–	–
20–05	S	S	S	S	S	**S**	–	–	–	–
20–06	S	S	S	S	S	S	**S**	–	–	–
20–07	S	S	S	S	S	S	S	**S**	–	–
20–08	S	S	S	S	S	S	S	S	**S**	–
Cubicle 2										
20–09	S	**S**	–	–	–	–	–	–	–	–
20–10	S	S	**S**	–	–	–	–	–	–	–
20–11	S	S	S	**S**	–	–	–	–	–	–
20–12	S	S	S	S	**S**	–	–	–	–	–
20–13	S	S	S	S	S	**S**	–	–	–	–
20–14	S	S	S	S	S	S	**S**	–	–	–
20–15	S	S	S	S	S	S	S	**S**	–	–
20–16	S	S	S	S	S	S	S	S	**S**	**S**
Contact animals										
20–17	S	S	S	S	S	S	S	S	S	**S**
20–18	S	S	S	S	S	S	S	S	**S**	–
20–19	S	S	S	S	**S**	–	–	–	–	–

*Bold indicates necropsy. Underline indicates introduction of contact animal to cubicle 1 or 2. ID, identification; S, sample collected; SARS-CoV-2, severe acute respiratory syndrome coronavirus 2; –, no sample collected.

### Animal Sampling

Oral, rectal, and nasal swab specimens were taken from each pig under general anesthesia by using isoflurane. Samples were placed into sterile D-PBS containing streptomycin, vancomycin, nystatin, and gentamycin. Fluid was collected from a bilateral nasal wash by using sterile D-PBS. Blood was collected in serum, sodium citrate, sodium heparin, and K3 EDTA collection tubes via jugular venipuncture.

### Hematology, Chemistry, and Blood Gas Analyses

Hematology was performed on an HM5 analyzer (Abaxis, https://www.abaxis.com) by using K3 EDTA-treated whole blood. We evaluated erythrocytes, hemoglobin, hematocrit, mean corpuscular volume, mean corpuscular hemoglobin, mean corpuscular hemoglobin concentration, red cell distribution weight, platelets, mean platelet volume, leukocytes, and absolute and percent neutrophil count, lymphocyte count, monocyte count, eosinophil count, and basophil count. Blood chemistries were evaluated on a VetScan 2 (Abaxis) with Comprehensive Diagnostic Profile rotors (Abaxis) by using serum stored at −80°C until tested. We evaluated glucose, blood urea nitrogen, creatinine, calcium, albumin, total protein, alanine aminotransferase, aspartate aminotransferase, alkaline phosphatase, amylase, potassium, sodium, phosphate, chloride, globulin, and total bilirubin. We used sodium heparin-treated blood to analyze venous blood gases by using an iSTAT Alinity V (Abaxis) instrument with a CG4+ cartridge (Abaxis) to measure lactate, pH, total carbon dioxide, partial pressure carbon dioxide, partial pressure oxygen, soluble oxygen, bicarbonate, and base excess. We used age-specific values and the instrument reference intervals to establish normal ranges (*18*–*20*).

### Necropsy

Necropsy was performed after euthanasia via sodium pentobarbital overdose, confirmation of death, and exsanguination by femoral artery laceration. We collected tissue samples from skeletal muscle, abdominal fat, liver, spleen, pancreas, duodenum, jejunum, ileum, spiral colon, kidney, gastrohepatic and mesenteric lymph nodes, right cranial lung lobe, right middle lung lobe, right caudal lung lobe, left cranial lung lobe, left caudal lung lobe, trachea, heart, tracheobronchial lymph nodes, cervical spinal cord, meninges, cerebrum, cerebellum, brainstem, olfactory bulb, nasal turbinates, submandibular lymph nodes, tonsils, trigeminal ganglion, and the entire eye. From female animals, we collected the uterus and ovaries of the reproductive tract en bloc. We collected epiglottis and laryngeal folds from some animals. We split tissue samples between 10% neutral-buffered formalin and fresh tissue. We also collected cerebrospinal fluid, urine (when possible), vitreous, and bronchoalveolar lavage by using DMEM.

We fixed tissues in 10% neutral phosphate-buffered formalin. We routinely processed and sectioned tissue at 5 µm and stained with hematoxylin and eosin (HE) for histopathologic examination. We performed in situ hybridization on 5 μm paraffin-embedded formalin-fixed tissue sections by using RNAscope 2.5HD Detection Reagent Red kit and V-nCoV2019-S probe (Advanced Cell Diagnostics, http://rna.acdbio.com). Then, we counterstained sections with Gill’s 1 hematoxylin (Leica Biosystems, https://www.leicabiosystems.com), dried, and coverslipped.

We propagated SARS-CoV-2 isolate hCoV-19/Canada/ON-VIDO-01/2020 (GISAID accession no. EPI_ISL_425177), on Vero E6 cells in DMEM supplemented with 1% fetal bovine serum. We titrated virus by plaque assay and performed viral isolation as previously described (*21*,*22*).

### Tissue Homogenization and Virus Isolation

Weighed, frozen tissue sections in Precellys bead mill tubes (Bertin, https://en.esbe.com) were thawed, and we added D-PBS to make 10% (w/v) tissue homogenates. We processed tubes by using a Minilys personal tissue homogenizer (Bertin, https://www.bertin-instruments.com) and clarified by centrifugation at 2,000 × *g*. We used TriPure Reagent (Roche, https://www.roche.com) to inactivate clarified homogenates, swab specimens, and fluids collected from experimental animals and extracted RNA in duplicate. Samples positive by semiquantitative real-time RT-PCR (qRT-PCR) samples were tested for virus isolation through standard plaque assay on Vero E6 cells by using freshly prepared homogenates of frozen tissue.

### RNA Extraction

We extracted total RNA from cell culture and experimental samples, including nasal, oral, and rectal swab specimens; nasal washes; oral fluids; whole blood in sodium citrate; and tissues by using MagMax CORE Nucleic Acid Purification Kits (ThermoFisher Scientific, https://www.thermofisher.com) per manufacturer’s recommendation with the following modifications. In brief, we diluted samples in TriPure Reagent (Sigma-Aldrich, https://www.sigmaaldrich.com) at a 1:9 ratio and used this in place of the manufacturer’s lysis buffer for inactivation. We used 650 µL of TriPure-inactivated sample, 30 µL of binding beads, and 350 µL of kit-provided CORE binding buffer spiked with ARM-ENTERO (Asuragen, https://asuragen.com) enteroviral armored RNA, then single washes in both wash 1 and wash 2 buffers, and a final elution volume of 30 μL of kit-supplied elution buffer by using the automated MagMax Express 96 system running the KingFisher-96 Heated Script MaxMAX_CORE_KF-96 (ThermoFisher Scientific). The spiked enteroviral armored RNA was used as an exogenous extraction and reaction control.

### Detection of SARS-CoV-2

We performed qRT-PCR on all extracted samples by using primers and a probe specific for SARS-CoV-2 envelope (E) gene (*23*), including forward primer E_SARBECO_F1 (5′-ACAGGTACGTTAATAGTTAATAGCGT-3′); reverse primer E_SARBECO_R2 (5′-ATATTGCAGCAGTACGCACACA-3′); and probe E_SARBECO-P1 (5′-ACACTAGCCATCCTTACTGCGCTTCG-3′). We prepared master mix for qRT-PCR by using TaqMan Fast Virus 1-step Master Mix (ThermoFisher Scientific) according to manufacturer’s specifications by using 0.4 µmol of each E gene primer and 0.2 µmol of probe per reaction. Reaction conditions were 50°C for 5 min, 95°C for 20 s, and 40 cycles of 95°C for 3 s then 60°C for 30 s. Runs were performed by using a 7500 Fast Real-Time PCR System (Thermofisher, ABI), and semiquantitative results were calculated based on a gBlock (Integrated DNA Technologies, https://www.idtdna.com) standard curve for SARS-CoV-2 E gene. For confirmation, we used SARS-CoV-2–specific primers targeting the spike (S) gene and the RNA-dependent RNA polymerase (RdRp) gene. For S, we used the forward primer SARS2_Spike_FOR (5′-TGATTGCCTTGGTGATATTGCT-3′); the reverse primer SARS2_Spike_REV (5′-CGCTAACAGTGCAGAAGTGTATTGA-3′); and the probe SARS2_Spike_Probe (5′-TGCCACCTTTGCTCACAGATGAAATGA-3′). For RdRp, we used forward primer RdRp_SARSr-F (5′-GTGARATGGTCATGTGTGGCGG-3′); reverse primer RdRp_SARSr-R (5′-CARATGTTAAASACACTATTAGCATA-3′); and probe RdRp_SARSr-P2 (5′-CAGGTGGAACCTCATCAGGAGATGC-3′). We tested all samples in duplicate and considered cycle threshold (C_t_) <36 positive.

### Genome Sequencing

We were able to extract SARS-CoV-2 RNA from the submandibular lymph node of 1 pig (20–06), which was processed for high-throughput sequencing by CFIA National Centre for Foreign Animal Disease (NCFAD) Genomics Unit with enrichment for sequences for vertebrate viruses according to previously published method (*24*,*25*) and sequenced on a MiSeq (Illumina, https://www.illumina.com) using MiSeq Reagent Kit v3 (600-cycle; Illumina). Data analysis also were performed by the CFIA NCFAD Genomics Unit using nf-villumina version 2.0.0 (https://github.com/peterk87/nf-villumina), an in-house workflow developed by using Nextflow (*26*), which performed read quality filtering with fastp (*27*); Centrifuge version 1.0.4-beta (*28*) and Kraken2 version 2.0.8 (*29*) read taxonomic classification using an index of the National Center for Biotechnology Information (NCBI) nucleotide database (downloaded 2020 Feb 4); a Kraken2 index of NCBI RefSeq (https://www.ncbi.nlm.nih.gov/refseq) sequences of archaea, bacterial, viral, and human genomes GRCh38 (downloaded and built on 2019 Mar 22); removal of nonviral reads (i.e., not classified as belonging to superkingdom “Viruses” (NCBI taxonomic identification 10239) by using Kraken2 and Centrifuge taxonomic classification results; de novo metagenomics assembly of taxonomically filtered reads by Shovill version 1.0.9 (*30*), Unicycler version 1.0.9 (*31*), and Megahit version 1.2.9 (*32*); and nucleotide BLAST+ version 2.9.0 (*33*,*34*) search of all assembled contigs against the NCBI nucleotide BLAST database (downloaded 2020 April 10) using the “update_blastb.pl” script as part of the blast Bioconda package (*35*). We mapped nf-villumina taxonomically filtered reads against the top viral nucleotide BLAST match, SARS-CoV-2 isolate 2019-nCoV/USA-CA3/2020, MT027062.1, to generate a majority consensus sequence.

### Serum Neutralization Assays

We determined neutralizing antibody titers in serum samples by using a plaque reduction neutralization test (PRNT) against SARS-CoV-2. Serial 5-fold dilutions of heat inactivated (30 min at 56°C) serum samples were incubated with virus for 1 h at 37°C. Each virus–serum mixture was then added to duplicate wells of Vero E6 cells in a 48-well format, incubated for 1 h at 37°C, and overlaid with 500 μL of 2.0% carboxymethylcellulose in DMEM per well. Plates were then incubated at 37°C for 72 h, fixed with 10% buffered formalin, and stained with 0.5% crystal violet. Serum dilutions with >70% reduction of plaque counts compared with virus controls were considered positive for virus neutralization. We used negative serum samples plus virus controls to estimate the percent reduction.

### Surrogate Virus Neutralization Test

Detection and semiquantitation of neutralizing antibodies were determined by using SARS-CoV-2 Surrogate Virus Neutralization Test Kit (Genscript, https://www.genscript.com) according to the manufacturer’s instructions. All samples from 7–29 dpi were assessed, including archived negative serum samples and kit-supplied negative controls. We considered values above the manufacturer’s recommended cutoff of 20% positive for neutralization.

## Results

Starting at 1 dpi, a mild, bilateral ocular discharge developed in the 16 experimentally inoculated pigs; in some cases, this discharge was accompanied by serous nasal secretion. Discharge was observed for only the first 3 dpi. Temperatures among pigs remained normal throughout the study (Appendix Table 1). Overall, animals did not develop clinically observable respiratory distress; however, 1 animal (pig 20–06) had mild depression with a cough at 1 dpi, which continued through 4 dpi. This animal did not display additional clinical signs over the course of the study.

Viral shedding can occur through droplets from coughing, sneezing, oral fluids, or gastrointestinal involvement. Thus, we developed a sampling schedule to determine the incidence of viral shedding (Table 1). Starting at 3 dpi, we sampled oral, nasal, and rectal swabs every other day up to 15 dpi, in case of delayed onset (*1*). We extracted nucleic acid from swabs and performed qRT-PCR to identify SARS-CoV-2 by targeting the E gene, but we did not detect viral RNA in swabs from any animals over the course of the study (Table 2).

**Table 2:** Detection of SARS-CoV-2 by real-time reverse transcription PCR of samples from experimentally inoculated pigs*

Samples	Days postinoculation									
	0	3	5	7	9	11	13	15	22	29
Swab specimens	0/57	0/57	0/51	0/45	0/39	0/30	0/24	0/18	0/12	0/6
Nasal wash	0/16	**2/16**	0/14	0/12	0/12	0/10	0/8	0/6	0/4	0/2
Blood	0/16	0/16	0/14	0/12	0/12	0/10	0/8	0/6	0/4	0/2

*Results are reported as number positive/number of samples collected. Bold text indicates clinically significant findings. SARS-CoV-2, severe acute respiratory syndrome coronavirus 2.

Nasal washes are a sensitive method for detection of pathogens in swine, and we routinely sampled nasal washes by using sterile D-PBS to rinse nasal passages. Two pigs (20–10 and 20–11) displayed low levels of viral RNA by qRT-PCR at 3 dpi (Table 2). We attempted recovery of live virus from PCR-positive nasal wash samples, but neither produced cytopathic effect or increased RNA detection via qRT-PCR of the cell culture supernatant.

We also used a noninvasive, group sampling method to evaluate viral shedding. A cotton rope was hung in animal pens before feeding; when pigs chewed on the rope, they deposited oral fluids. We processed fluids from ropes daily and samples from cubicle 1 had a weak positive signal for viral RNA at 3 dpi by qRT-PCR (Tables 1,3). We attempted virus isolation from this sample but were not able to isolate the virus. Of note, the positive oral fluid did not come from the same room as the 2 positive nasal washes from pig 20–10 and pig 20–11, which were housed in cubicle 2. Therefore, at least 3 animals provide evidence of viral nucleic acid in oronasal secretions from 2 independent animal rooms. In addition, we did not detect viral infection at any point from the 2 naive transmission contact pigs introduced to the infected pigs at 10 dpi.

**Table 3:** Detection of SARS-CoV-2 by real-time reverse transcription PCR of oral fluid samples from experimentally inoculated pigs*

dpi	No. samples tested	No. samples positive
0	2	0
1	2	0
2	2	0
3	2	**1**
4	2	0
5	2	0
6	2	0
7	2	0
8	2	0
9	2	0
10	2	0
11	2	0
12	2	0
13	2	0
14	2	0
15	2	0
16	2	0
17	2	0
18	2	0
19	2	0
20	2	0
21	2	0
22	1	0
23	1	0
24	1	0
25	1	0
26	1	0
27	1	0
28	1	0
29	1	0

*Oral fluid samples were collected from shared chew toys placed in enclosures for animal enrichment. Bold text indicates clinically significant results. dpi, days post inoculation; SARS-CoV-2, severe acute respiratory syndrome coronavirus 2.

After collecting samples, we attempted SARS-CoV-2 detection from whole blood by qRT-PCR (Table 1). Viremia, as indicated by the presence of viral RNA in the blood, was not detected in any animal during the study (Table 2). We measured blood cell counts by using the VetScan HM5 (Abaxis), blood chemistries by using VetScan 2 (Abaxis), and blood gases by using i-STAT (Abbott, https://www.abbott.com). Although some laboratory variation was observed during the study, changes were minimal and inconclusive, and profiles consistent with acute viral infection or subsequent organ damage were not observed.

To identify potential target tissues or gross lesions consistent with SARS-CoV-2, we performed necropsy on 2 animals every other day from 3 dpi through 15 dpi and necropsied an additional 2 pigs at both 22 dpi and 29 dpi (Table 1). No clinically significant pathology was observed that could be attributed directly to a viral infection. We performed qRT-PCR across all tissues and samples collected at necropsy targeting the E gene of SARS-CoV-2 (Table 4). One tissue sample, the submandibular lymph node from pig 20–06, necropsied at 13 dpi, was positive by qRT-PCR (C_t_ = 32) for viral RNA. The tissue sample testing was repeated in triplicate, on independent days, and generated consistent results. Further, RNA was extracted from homogenized tissue, and we recovered the full genome sequence of SARS-CoV-2 from pig 20–06.

**Table 4:** Detection of SARS-CoV-2 by real-time reverse transcription PCR of tissue samples from experimentally inoculated pigs*

Pig ID	dpi	No. samples tested	No. samples positive
20–01	3	35	0
20–09	3	35	0
20–02	5	36	0
20–10	5	35	0
20–03	7	35	0
20–11	7	35	0
20–04	9	35	0
20–12	9	34	0
20–05	11	35	0
20–13	11	36	0
20–06	13	35	**1**
20–14	13	35	0
20–07	15	36	0
20–15	15	35	0
20–08	22	35	0
20–18	22	35	0
20–16	29	35	0
20–17	29	35	0
*Tissue samples were collected from pigs during necropsy from skeletal muscle, abdominal fat, liver, spleen, pancreas, duodenum, jejunum, ileum, spiral colon, kidney, gastrohepatic and mesenteric lymph nodes, right cranial lung lobe, right middle lung lobe, right caudal lung lobe, left cranial lung lobe, left caudal lung lobe, trachea, heart, tracheobronchial lymph nodes, cervical spinal cord, meninges, cerebrum, cerebellum, brainstem, olfactory bulb, nasal turbinates, submandibular lymph nodes, tonsils, trigeminal ganglion, and the entire eye. Bold text indicates clinically significant results. dpi, days postinoculation; ID, identification; SARS-CoV-2, severe acute respiratory syndrome coronavirus 2.			

We generated a 10% homogenate from the submandibular lymph node of pig 20–06 and used it to infect Vero E6 cells. We took aliquots from the cell culture on days 2 and 3 postinfection to monitor viral replication as indicated by an increasing quantity of RNA. On day 3, we observed mild cytopathogenic effect in the first passage with an increase in viral RNA measured by qRT-PCR targeting the E, S, and RdRp genes. The first passage supernatant was clarified by centrifugation and a second passage performed in Vero E6 cells. At day 2 postinfection of the second passage, we observed substantial cytopathogenic effect and increasing copies of SARS-CoV-2 viral RNA, confirmed by qRT-PCR. Together, these findings demonstrated the presence of live, replication-competent SARS-CoV-2 virus isolated from the submandibular lymph node of pig 20–06 (Table 2).

We monitored development of SARS-CoV-2 neutralizing antibodies over the course of study. Starting at 7 dpi, we obtained serum from individual animals for both virus neutralization test (VNT) and a surrogate VNT (sVNT) using cPass Neutralization Antibody Detection kit (Genscript, https://www.genscript.com). Serum samples first were tested by using a traditional VNT; 1 pig (20–07) generated neutralizing antibody titers, albeit weak, at a 1:5 dilution with a 70% reduction of plaques at both 13 dpi and 15 dpi (Table 5). Consequently, the sVNT assay identified the same animal, pig 20–07, as antibody-positive with 0.188 µg/mL antibody at 15 dpi. A second pig (20–14) generated antibodies at 11 dpi (0.113 µg/mL) and 13 dpi (0.224 µg/mL). We also used sVNT to identify secreted antibody in oral fluids. Of note, at 6 dpi we detected positive antibody (0.133 µg/mL) from group oral fluid collected from cubicle 1 (Table 5).

**Table 5:** Neutralizing antibody development in serum and oral fluids from pigs experimentally inoculated with SARS-CoV-2*

Pig ID	Serum samples, dpi, VNT/sVNT

0			7			9		11			13			15			22			29		
20–01	–/–			–/–			–/–		–/–			–/–			–/–			–/–			–/–		
20–02	–/–			–/–			–/–		–/–			–/–			–/–			–/–			–/–		
20–03	–/–			–/–			–/–		–/–			–/–			–/–			–/–			–/–		
20–04	–/–			–/–			–/–		–/–			–/–			–/–			–/–			–/–		
20–05	–/–			–/–			–/–		–/–			–/–			–/–			–/–			–/–		
20–06	–/–			–/–			–/–		–/–			–/–			–/–			–/–			–/–		
20–07	–/–			–/–			–/–		–/–			**+/–**			**+/0.188**			–/–			–/–		
20–08	–/–			–/–			–/–		–/–			–/–			–/–			–/–			–/–		
20–09	–/–			–/–			–/–		–/–			–/–			–/–			–/–			–/–		
20–10	–/–			–/–			–/–		–/–			–/–			–/–			–/–			–/–		
20–11	–/–			–/–			–/–		–/–			–/–			–/–			–/–			–/–		
20–12	–/–			–/–			–/–		–/–			–/–			–/–			–/–			–/–		
20–13	–/–			–/–			–/–		–/–			–/–			–/–			–/–			–/–		
20–14	–/–			–/–			–/–		**–/0.113**			**–/0.224**			–/–			–/–			–/–		
20–15	–/–			–/–			–/–		–/–			–/–			–/–			–/–			–/–		
20–16	–/–			–/–			–/–		–/–			–/–			–/–			–/–			–/–		
20–17	–/–			–/–			–/–		–/–			–/–			–/–			–/–			–/–		
20–18	–/–			–/–			–/–		–/–			–/–			–/–			–/–			–/–		
20–19	–/–			–/–			–/–		–/–			–/–			–/–			–/–			–/–		
Oral sample collection, dpi†																							
All	0	1	2		3	4		5		6	7		8	9		10	11		12	13		14	15
	–	–	–		–	–		–		**0.113**	–		–	–		–	–		–	–		–	–

*Individual serum samples were collected from pigs; oral fluid samples were collected from shared rope chews placed in enclosures. VNT is measured 1:5; sVNT is measured µg/mL. Bold text indicates results with clinical significance. dpi, days post inoculation; sVNT, surrogate virus neutralization test; VNT, virus neutralization test; –, no detectable neutralizing antibodies; +, neutralizing antibodies detected. †sVNT only.

## Discussion

Our study found that domestic swine are susceptible to low levels of SARS-CoV-2 viral infection. Among 16 experimentally inoculated animals, 5 (31.3%) displayed some level of exposure or elicited an immune response to the virus. Only 1 pig in our study retained live virus, but 2 other animals had detectible RNA measured in nasal wash, and another 2 developed antibodies. One pig (20–06) displayed mild, nonspecific clinical signs, including coughing and depression. Then, over the 9 days between cessation of clinical signs and postmortem evaluation, we found this pig maintained the virus in the submandibular lymph node, but virus was undetected in other samples from this animal. In addition, multiple pigs demonstrated mild ocular and nasal discharge that appeared during the immediate, postinfection period. Of note, among 5 animals with potential infection, we detected only low levels of viral RNA; no live viral shedding was identified.

After detection of viral RNA in group oral fluids collected by rope chews at 3 dpi, we detected secreted antibody by using sVNT; we detected viral RNA in the same sample type at 6 dpi. The amount of antibody measured in oral fluids from swine would be considered below a protective cutoff based on comparisons to classical neutralizing titers, however the discovery of secreted antibody in oral fluids might be useful for surveillance efforts. This finding also demonstrates the possibility that human saliva should be evaluated as a less invasive method to provide accompanying evidence with serosurveillance studies for exposure to SARS-CoV-2.

The results of this study contradict previous reports indicating swine are not susceptible to SARS-CoV-2 infection (*4*,*36*). Previous studies did not detect RNA in swabs or organ samples, and no seroconversion was measured. Infectious dose, viral isolate, age, and breed or colony of swine could affect study outcomes. Of note, we used a 10-fold higher viral dose for experimental infection than was used in previous studies. Moreover, we obtained animals from a high health status farm in Manitoba, rather than a specific pathogen–free colony, to determine the risk to farmed pigs in Canada. Altogether, these findings indicate that further investigations into the susceptibility of additional domestic livestock species should be conducted to assess their risk for infection and zoonoses. Finally, we emphasize that to date no SARS-CoV-2 cases among domestic livestock have been documented by natural infection; however, the results of this study support further investigations into the role that animals might play in the maintenance and spread of SARS-CoV-2.

This article was preprinted at https://biorxiv.org/cgi/content/short/2020.09.10.288548v1.

Appendix Additional information on susceptibility of domestic swine to experimental infection with SARS-CoV-2.
